# Synergies, strengths and challenges: findings on community capability from a systematic health systems research literature review

**DOI:** 10.1186/s12913-016-1860-1

**Published:** 2016-11-15

**Authors:** Asha S. George, Kerry Scott, Vrinda Mehra, Veena Sriram

**Affiliations:** 1 Department of International Health, Johns Hopkins Bloomberg School of Public Health, Baltimore, MD USA; 2 South African Research Chair in Health Systems, Complexity and Social Change, School of Public Health, University of Western Cape, Cape Town, South Africa; 3 Global health consultant, Bangalore, India

## Abstract

**Background:**

Community capability is the combined influence of a community’s social systems and collective resources that can address community problems and broaden community opportunities. We frame it as consisting of three domains that together support community empowerment: *what communities have; how communities act;* and *for whom communities act.* We sought to further understand these domains through a secondary analysis of a previous systematic review on community participation in health systems interventions in low and middle income countries (LMICs).

**Methods:**

We searched for journal articles published between 2000 and 2012 related to the concepts of “community”, “capability/participation”, “health systems research” and “LMIC.” We identified 64 with rich accounts of community participation involving service delivery and governance in health systems research for thematic analysis following the three domains framing community capability.

**Results:**

When considering *what communities have,* articles reported external linkages as the most frequently gained resource, especially when partnerships resulted in more community power over the intervention. In contrast, financial assets were the least mentioned, despite their importance for sustainability. With *how communities act,* articles discussed challenges of ensuring inclusive participation and detailed strategies to improve inclusiveness. Very little was reported about strengthening community cohesiveness and collective efficacy despite their importance in community initiatives. When reviewing *for whom communities act,* the importance of strong local leadership was mentioned frequently, while conflict resolution strategies and skills were rarely discussed.

Synergies were found across these elements of community capability, with tangible success in one area leading to positive changes in another. Access to information and opportunities to develop skills were crucial to community participation, critical thinking, problem solving and ownership. Although there are many quantitative scales measuring community capability, health systems research engaged with community participation has rarely made use of these tools or the concepts informing them. Overall, the amount of information related to elements of community capability reported by these articles was low and often of poor quality.

**Conclusions:**

Strengthening community capability is critical to ensuring that community participation leads to genuine empowerment. Our simpler framework to define community capability may help researchers better recognize, support and assess it.

## Background

### Rationale

Communities are a vital and vibrant part of health systems. They form the social boundaries that define the individuals and households whose health outcomes matter as a health systems goal, but also the social context for the relationships that underpin the success of many health systems interventions. An extensive literature reviews the extent and effectiveness of community participation for health [1–4]. Consensus emerging from this literature emphasizes the need to further understand the underlying social processes and conditions enabling communities to engage effectively with health systems to improve their health. These underlying social processes and conditions include community capability.

Community capability is the combined influence of a community’s social systems and collective resources that can be applied to address community problems and broaden community opportunities. Norton et al. define it as “a set of dynamic community traits, resources, and associational patterns that can be brought to bear for community building and community health improvement” [5]. Since the late 1990s, various attempts have been made to define community capability [6–9] some of which aim to identify domains that can be measured quantitatively [10] and apply it to improving sexual, reproductive, maternal and child health programs [4, 11, 12].

With an aim to create a comprehensive account of the elements that constitute community capability, Liberato et al. undertook a review of community capacity building, development and participation and identified nine domains and six sub-domains, some of which showed more consensus in the literature than others [13]. While some domains reflect both inputs and outcomes of community capacity (existence and sharing of community assets, robustness of social cohesion and collective efficacy), other domains reflect the processes and governance of power relations within communities (social participation, political voice, critical thinking skills, conflict resolution skills, leadership/champions). A key observation of the review was that a large part of the community capability literature comes from high and middle income country contexts [13].

In contrast, a parallel literature grounded in low income country settings and inspired by Amartya Sen [14] theorizes that the contribution of ‘capabilities’ defines what societies must do to ensure individual functioning and substantive freedoms. It is argued that such capabilities are a central part of implementing health interventions sustainably and equitably, as well as supporting development more broadly. A key distinction is that while agency and freedoms are valued, capabilities are not seen exclusively within the remit of communities to develop. There is recognition of broader societal responsibility to support such capabilities.

**Fig. 1:**
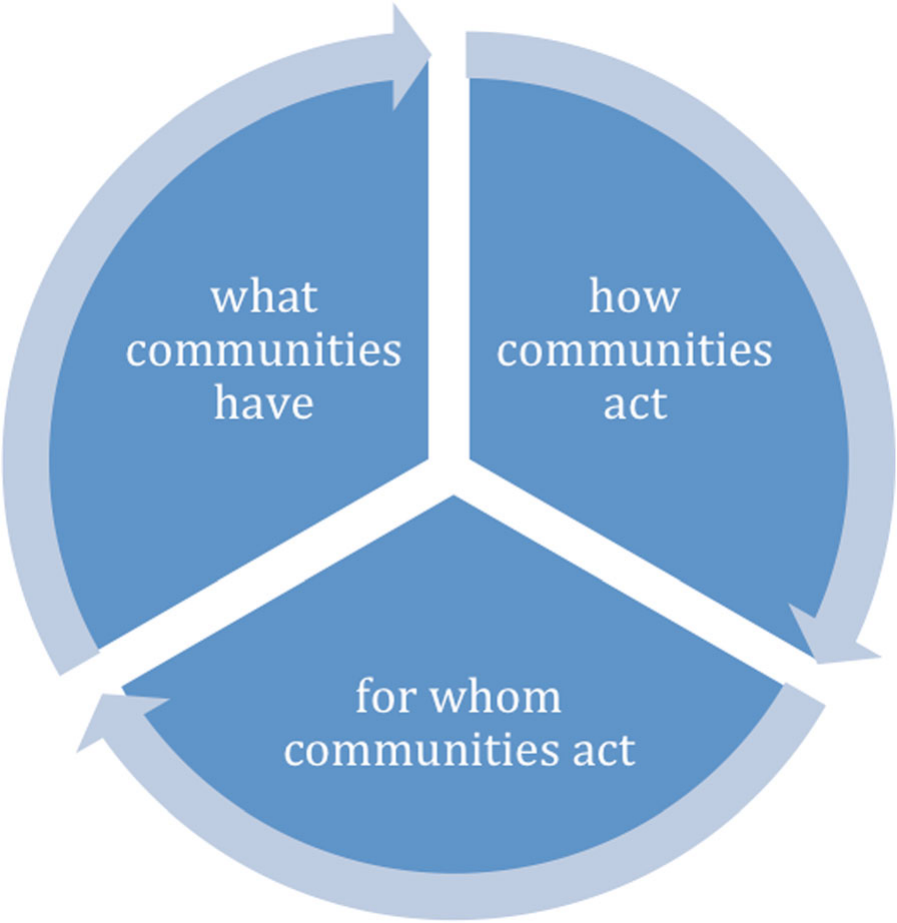
Community capability: A synergy of what communities have, how they act and for whom they act

Drawing from these various bodies of work, we defined community capability as consisting of three domains which synergistically support community empowerment: *what communities have; how communities act,* and *for whom communities act* (Fig. 1). These domains encompass material assets and resources, including information and skills, that communities must have to support collective endeavors. In addition, it takes into account the governance processes and characteristics that support both how assets and resources are shared and controlled, how communities function collectively and the interests served by the community’s collective action and social processes. Each of these domains is necessary but not sufficient on its own to ensure that communities are empowered to improve their health and well-being. Table 1 clusters the many aspects of community capability we identified in the literature under the three domains of our community capability framework.

**Table 1:** Domains framing the elements of community capability

What communities have	How communities act	For whom communities act
Physical assets^a,c,d^	Inclusiveness^a^	Critical thinking^a,c,d^
Financial assets^a,b,d^	Cohesiveness^b,d^	Voice^a^
Information^a,d^	Efficacy^b,d^	Leadership^a,b,c,d^
Skills^a,d,e^	Sense of community^a,d^	Conflict solving^a,b^
External links^a,c,d^	Networking^a,c,d^	Critical reflection^a,d^
Community values^d^	Participation^b,c,d^	Culture of openness^d^
Control^c^	Partnership building^a,d^	Participatory decisionmaking^a^
Vision^d^	Commitment to action^a,d^	Institutions and organizational structures^a,c,d^
Community history^d^	Resilience^d^	

^a^Liberato, et al. 2011 [13]; ^b^Underwood, et al. 2012 [11]; ^c^Gibbon, et al. 2002 [9]; ^d^Vijayaraghavan, 2007 [95]

### Objectives

Drawing from different bodies of work describing community capability, we created a framework simplifying the relationships between elements of community capability, and sought to understand these relationships through health systems intervention research in LMICs that supported community participation. Participants included community members involved on a collective basis in health systems interventions in LMICs. As this was largely a qualitative review, specific comparison interventions or populations were not sought and a broad array of study designs were considered eligible, whether experimental, descriptive or exploratory/explanatory.

## Methods

We undertook a secondary analysis of the documents identified through our previous systematic review on the nature of community participation in health systems interventions in low and middle income country contexts [2]. The database was constructed by searching for literature published from January 2000 to June 2012 in four electronic databases: Pubmed, Embase, Scopus/Web of Science, Global Health (Ovid) related to the concepts of “community”, “capability/participation”, “health systems research” and “LMIC”. Our search generated 3,803 articles, which, after removing 711 duplicates, left a total of 3,092 articles. Following study criteria (Table 2), iterative rounds of screening abstracts and full texts with peer review weekly discussions led to 260 studies with some level of community participation in health systems research studies.

**Table 2:** Inclusion and exclusion criteria

Inclusion	Exclusion
1. Health systems research which examines an interaction of parts (service delivery, information systems, medical products/ technologies, human resources, financing, governance, community/ households) and their interconnections (ideas and interests, relationships and power, values and norms) that come together for a purpose (health) 2. Low and middle income country (LMIC) contexts 3. 2000 onwards 4. English language publication, with American and English spellings 5. Peer review journals 6. Community level health system interventions where communities were substantially involved in implementation or monitoring and evaluation, i.e., going beyond initial consultations for design or formative research. Community was defined as people residing together in a geographical area, a village or a township, because social boundaries are most often manifested in geographic areas, particularly in LMICs. However, articles involving communities organized around a particular health condition but that were not geographically defined were also included. Communities did not include community based organizations and or local administrators who worked in these geographic areas but resided outside them.	1. Basic scientific research, clinical efficacy or effectiveness of treatments/ technologies, measurement and social determinants of population health 2. Editorials 3. Review papers will not be abstracted through the form, but will be reviewed as background material.

A subset of 64 of these studies were found to be rich accounts of community participation involving service delivery and governance in health systems research, the focus of our interest. Rich accounts of community participation was assessed by combining the number of elements of intervention research that a community would be involved in (identifying and defining problems; identifying and defining interventions; implementing interventions; managing resources for intervention; and monitoring and evaluating interventions) with the level of detail available on community participation in the article. Those categorized as rich participation largely correlate with the increasing number of elements. However, articles that may have only supported community participation in one or two elements of the intervention but provided a rich description of this participation whether positive or negative where included, rather than those that had more than one element but with little description detailing what this meant for the communities involved.

Based on our framework, community capability elements that we report on include: what communities have (physical and financial assets, information and skills, external linkages), how communities act (breadth of participation, cohesiveness and efficacy), and for whom communities act (leadership, conflict solving). These were the elements that were most frequently cited in the literature and where at least some information was found in our database. We did not seek to analyze all elements of community capability listed in Table 1. Findings were synthesized using a thematic approach, commonly used to summarize qualitative and quantitative studies in systematic reviews [15, 16]. While numbers are cited in some instances, we came to recognize that the elements we analyzed are social processes that have nuanced interpretations and contextual variations that were not captured by simple counts. Moreover, as noted in our limitations section the quality of documentation of these social processes was also weak. We therefore felt it was inappropriate to hinge our analysis on numerical counts. Articles were revisited multiple times and abstracted findings synthesized into detailed outputs. These were then reviewed and revised by the lead author (AG) in discussion with the team, following a process of constant comparison. After drafting synthesized findings, authors revisited original articles to check their interpretations.

## Results

### What communities have

A key part of community capability are the resources that communities have supporting their empowerment and better health. These include physical and financial assets, information/skills and linkages to external actors. In our review, about four-fifths of the 64 service delivery and governance articles with rich experiences of community participation mentioned these resources. Most articles discussed, in order of frequency, external links, gaining skills and information, and accruing physical assets. Relatively fewer articles referred to the development of and control over financial assets as a key resource supporting community capability. In this section, we review in further detail key thematic findings from the review about these elements of community capability related to what communities have.

#### Physical and financial assets

In terms of physical assets, articles detailed improvements at the community level of medical infrastructure and supplies, in addition to broader types of infrastructural development including water tanks, sanitation pits, hand pumps, public toilets and transport systems. Although mentioned to a lesser degree, articles also mentioned financial assets gained through income generation and microfinance/microcredit projects. Income generating activities ranged from small scale efforts, such as weaving mats, cotton blankets, embroidery, small household shops and raising pigs, to large scale projects that installed grain mills and widely improved agricultural practices. Articles in the review mentioned these physical and financial assets in a descriptive fashion, with few delving into issues of whether communities had control over these resources and how they were governed.

Some articles detailed synergies between the creation of physical or financial assets and other community activities and abilities [17–20]. For example, in Tanzania, the experience of working collectively on community-based transport systems for pregnant women led village health workers to organize themselves into professional groups and associations that then pooled funds to begin a micro-credit association for themselves [17].

While not documented extensively, a few articles inferred that having an indigenous financing mechanism facilitated the continuation of an intervention once initial seed money or funding ended [18, 21, 22]. Examples of local financial resource mobilization included charging small fees to support service delivery [21, 23, 24], equity fund collection boxes alongside those used for daily operations or voluntary community donations [18]. Articles also documented the converse: setbacks due to lack of resources and support [25–28]. For example, a community based rehabilitation program in Vietnam recognized financing as an essential condition for a sustainable program. However, project staff, village health workers, persons with disabilities and their families, voiced that the program failed to give sufficient information on how financial sustainability could be achieved and felt dependent on external support [28].

#### Information and skills

Several articles detailed how communities acquired information pertaining to a range of health topics, and developed skills related to problem solving and management.

Articles described the acquisition of information on health topics ranging from maternal health, family planning, HIV, nutrition, hygiene and the treatment of certain diseases such as onchocerciasis. While some articles detailed community wide media campaigns, many focused on the role of community volunteers, peers and workers supporting counseling, peer education and in certain instances basic clinical skills related to danger signs, side effects of drugs and drug dosage.

Articles to a lesser extent documented sharing information with community members on issues related to implementation of the intervention, the nature of community participation involved, the extent of entitlements or rights to be claimed, or information on broader social and contextual factors. A few articles discussed how *not* providing such information was a major barrier to successfully meeting goals [29–31]. For example, an examination of HIV prevention programs that included youth participation in a South African township found that critical thinking regarding the contextual factors underlying sexual behavior was not actively encouraged among the project’s peer educators and therefore, these individuals were not able to adequately support youth with context-specific behavior change communication [31].

Overall interventions were more likely to disseminate information related to community participation and broader health and societal issues if their focus was beyond specific health conditions, and more centered on health systems strengthening more broadly [18, 32]. However, several articles were exceptions to this overall pattern as they related to specific health conditions but also disseminated information supporting broader community participation. These included articles focusing on mental health [33], onchocerciasis control [24, 34, 35], participatory learning and action cycles through women’s groups in India and Nepal to improve maternal and newborn health [19, 20, 36], and efforts by community-based organizations in India to support to HIV-positive individuals to access government entitlement programs [37].

Several interventions focused on skills development among community members, ranging from service delivery to project management. Several articles documented the development of problem solving and managerial skills such as planning, implementation, budgeting and leadership [17, 22, 36, 38–42]. For example, women’s groups in India, Nepal and Bangladesh engaged community members in prioritization of health problems and planning of strategies to address maternal and newborn health problems [20, 36, 43, 44]. In addition, some articles documented training community members in monitoring and evaluation [43, 45–47] and others helped to build community communication and advocacy skills [33, 36, 48].

Developing these kinds of skills was seen as aiding community ownership and therefore sustainability. The premise of community directed treatment programs supported by African Program for Onchocerciasis Control (APOC) was that the target communities assume full ownership and responsibility for planning, implementing, overseeing control of Onchocerciasis and emerge as a lead stakeholder [24, 34, 35]. This strategy, launched in the mid-1990s, was sustained over a 20 year period to reach 100.79 million people by 2013 and is estimated to have decreased the number of people infected from 37.9 million in 1995 to 15.1 million in 2011 [49]. In Tanzania, the development and implementation of community-based action plans for emergency transportation for pregnant women was centered around the idea that community members must take the lead in decision making, and emphasized the participation of women in these processes [41]. Five years after project initiation, 13 of the 50 intervention communities had functional emergency transportation plans, run on local resources [41]. Community ownership ensured that the village health workers trained in this project continued to provide an array of reproductive health services, referral and counseling 6 years after the formal completion of the project [50].

#### External linkages

Relationships with multiple stakeholders such as central and local health authorities, international and national NGOs and other organizations were listed as the most frequent resource gained by communities, although the quality of information detailing the nature of those linkages was poor. External linkages had three broad and frequently overlapping purposes: to increase delivery and utilization of services, to improve accountability of services, and to support higher level planning.

Many of the linkages that focused on strengthening relationships between healthcare providers, NGOs and community members did so to support community delivery of interventions (such as Directly Observed Treatment Short course (DOTS) or ivermectin), community uptake of health services, and community education through involving health workers as experts in community forums [35, 51–55]. There were both positive and negative examples of facilitating these linkages to support the delivery and utilization of services. Mushi [51] describes the supportive relationship between Safe Motherhood Promoters and health providers wherein the promoters felt valued, supported and welcome in health facilities. As mentioned earlier, MacPhail & Campbell [31] described the successful linkage created between a youth friendly health facility in South Africa and the national HIV program loveLife, which enabled youth to access funding for their program. They contrast this successful linkage with the inability of school-based youth peer educators to access networks or alliances that could have supported them, because of their systematic exclusion from stakeholder committees [31].

As mentioned, some community linkages with local health authorities also sought to strengthen the accountability and quality of frontline health services. This was done through involving community members in management, oversight and supportive functions through committees and user associations [29, 38, 56, 57]. Linkages were also established between communities, health system actors and non-governmental actors to facilitate community input into higher-level program planning and decision-making [29, 42, 58]. While some papers reported significant positive collaborations arising from these latter two types of linkages [42, 58], others found difficulties [25, 29–31, 59].

Positive examples include an HIV prevention program in Lao, which successfully trained village youth volunteers in participatory research and analysis to inform district action plans; this village level analysis was used to influence policy through a youth network from the village all the way up to the central level [58]. Similarly, in China, the Women’s Reproductive Health and Development Program involved rural women in photovoice research and focus groups to identify the issues that they felt needed to be addressed through the intervention [42]. Local women then conveyed their needs to provincial and county guidance groups during program planning workshops. These guidance groups were composed of members of organizations and agencies involved in health, education, women’s wellbeing, economic development, and family planning. The authors attribute the project’s success to the establishment of these collaborative inter-agency groups, which linked to local women, thus enabling “bottom up” problem solving.

In contrast, Mosquera et al. [29] found that in Colombia, users association and customer service offices, which were created to channel citizen participation, failed to establish communication channels with the community they represented. In addition, members of user associations felt that they did not have adequate knowledge of the health system and participatory mechanisms to successfully influence decision-making. Furthermore, the authors found that policymakers and health care managers were not convinced that it was feasible or beneficial to involve users in technical and managerial matters [29].

While decentralization mandates often sought to improve community linkages to decision-makers, Harman [60] explored the dynamics that arose when it was a donor’s mandate that pushed for community involvement, without clear buy in from national governments. The World Bank’s HIV program in Kenya, Tanzania and Uganda sought to engage civil society organizations (CSOs) by funding their activities through national and district AIDS councils. However, this collaboration was tenuous as the district AIDS councils only agreed to work with CSOs to receive donor funding and were often unable to fund CSO proposals on time due to bureaucratic blockages. Furthermore, CSO competition for grants bred mistrust and undermined civil society networks and collective advocacy.

### How communities act

We drew information from articles regarding the characteristics of communities that influenced their ability to act collectively in the pursuit of a common goal, focusing on breadth of participation, cohesiveness, and efficacy. Most articles discussed breadth of participation (social inclusiveness) in contrast to cohesiveness and efficacy, the latter being attributes that evaluate the degree to which communities want to be a part of a group and work together towards a shared vision.

#### Breadth of participation

Examples of broad participation include those that engaged community members irrespective of their caste, gender or socio-economic differences, such as women from diverse backgrounds and various ethnic groups [19, 41, 42, 45, 56] or committees with representatives from different community based organizations and vulnerable groups [23, 33]. Some examples also detailed how initiatives spread beyond their intended target group, for example women’s groups in Malawi later formally included men [19, 20, 43, 61].

Breadth of participation was aided when decisions were made at public forums, community-wide meetings or community dialogues after consultation and consensus among community members [34, 41, 51, 56, 62, 63]. Some articles explicitly state that group meetings or classes were open to all, with no restriction on type of participants, and interventions took specific measures to ensure inclusion, such as offering scholarships to remove financial barriers to enrolment [22] or by not selectively mobilizing the elite or better-off groups [19, 22]. An intervention propagating a supervisory model for local health facilities restricted involvement to community leaders, but ensured representation from teachers, village headman, representatives from sub-district administration office, group of elders, housewives, village health volunteer groups and village development committee as leaders [38]. Other articles were explicit that anyone residing in the community, wanting to serve and fulfilling the selection criteria could be selected as a community based volunteer with no discrimination [51, 54].

Though there were a number of positive examples; there were more articles that detailed challenges to elicit wide community involvement. Women, youth, the less educated, the elderly and ethnic minorities were found to be excluded from decision making processes dominated by men, older people wealthier families and/or those from more powerful ethnic groups [30]. In other instances, selection processes for community representatives, volunteers or workers were politicized with lack of open communication [29] and limited channels for inputs by the wider community [59, 64–66].

Even if achieved, breadth of participation at times was not uniform. For example, in Nepal a women’s group intervention organized community level meetings to enable increased community participation in the planning process. But in nine places, communities were apathetic towards the group and did not want to commit and at four other places, the group met with hostility from community leaders [20]. Several studies observed varying levels of participation across activities [18, 46, 47, 67]. For example, the community-based reproductive health project in Tanzania mentioned earlier, reported that in 58 % of the communities sampled, women attended and participated in meetings but in 25 % of the communities, women attended meetings but did not necessarily participate in the discussion [17].

Recognizing these barriers, five articles discussed interventions that prioritized engaging the most marginalized people within the community. Some articles had an explicit focus on poor communities [18, 22, 31, 47], with one focused on addressing economic differences through subsidizing services or products [23]. Articles also focused on groups facing disadvantages due to belonging to minority and ethnic groups [45, 68] including those related to caste [55]. Measures to ensure fair representation included explicit inclusion of marginalized groups in self-help groups for internally displaced persons and repatriated returnees in Cambodia [45], inclusion of minority Cham Muslim representatives in Health Center Co-Management Committees in Cambodia [68], and ensuring caste diversity amongst community health volunteers in Kolkata, India [55] and Adivasi or tribal groups in women’s groups in Orissa, India [36].

Nonetheless, three studies discussed challenges with reaching the poorest households and communities, due to the design of the intervention. Tanaka, Kunii, Okumura and Wakai [53] examined refugee participation in an encamped health services program in Tanzania, and found that despite efforts to reach as many individuals as possible, those with less education and social support were found to be left out. Program planners (technical consultants and provincial policymakers) also excluded the poorest and more remote communities in a participatory women’s health intervention in China, due to the requirement of local funds to match donor commitments [42]. The authors of a study analyzing an urban health intervention in Zambia and Tanzania extrapolate that user fees in those study settings might have limited the utilization of public sector health facilities by the poorest households, and therefore, their participation in the user committees that made up the core of the interventions [69].

With regards to the explicit inclusion of women, some articles detailed examples women were the main active agents supporting the intervention and supported planning and advisory processes [28, 55, 56, 66, 70], or women were selected alongside men as community health workers or intervention facilitators [51, 52, 71]. Successful participation by women either hinged upon the support of the male members of their households [72, 73] or was observed in roles that were coherent with their culturally prescribed responsibilities such as input on maternal and child health [22, 24, 27]. In a study of decision making processes related to health services in Tanzania, Shayo, Norheim, et al. [65] found that women’s voices were particularly valued during decision-making around maternal health, but were not given the same consideration when discussing other health priorities. However, in some instances, women were mobilized to actively participate as the intervention was seen as an opportunity to build one’s self-confidence and extend beyond their traditional role as housewives [19, 22, 39, 55].

Certain articles highlighted the minimization or exclusion of women from decision-making forums such as community meetings [24, 27, 59]. In another example, Harpham and Few [64] note that despite efforts to prioritize the involvement of women on ‘health boards’ in Dar es Salaam, Tanzania, women’s representation did not meet the minimum requirement. A community-based onchocerciasis treatment program using community members as drug distributors found that there were fewer female community-directed health workers than males, and that women were less likely to be selected as community-directed health workers, despite support from community members [72].

In certain contexts, participation by women in decision making processes was either viewed as a “rebellion against authorities” [30] or were viewed as insufficient due to perceptions that women lacked the same abilities as men. For instance, with regards to community health planning, regardless of education status, women felt that they were undermined by men during decision-making due to their sex [65]. Traditionally, the social and legal systems in Ugandan communities restrict women from individually providing services beyond their families. In a community-directed treatment intervention with ivermectin, women covered a large area beyond their kinship, which put them in conflict with social legal systems, jeopardized their reputation and limited participation [74].

#### Cohesiveness and efficacy

Cohesiveness, a measure of community’s motivation and willingness to stay together as a group was discussed in a handful of articles [19, 22, 31, 53, 55, 58, 69, 74, 75]. A sense of serving ones’ community led to highly motivated community volunteers [34, 50, 64, 67]. In a community-directed treatment intervention with ivermectin in Uganda, community-drug distributors selected from kinship networks viewed their responsibilities in serving other kinsmen as a moral obligation [74].

In other instances, increased access to social networks, increased confidence [30, 38, 53, 75], skills, knowledge [38, 50]. For example with regards to community clinics and local health groups in Mexico, the groups’ gatherings, composed primarily of women, served as a therapeutic outlet for participants and a socially sanctioned place for them to socialize, learn and heal one another [22].

Efficacy, defined as the factors that enable communities to work together, was infrequently discussed and was mentioned in only 7 of the 64 articles discussing rich community participation related to service delivery and governance [31, 52, 57, 69, 74, 76, 77]. Key institutional processes that were supportive of efficacy that were described included how communities informally conducted meetings at specific intervals to identify problems, discuss health plans and implement programmatic decisions, and often formed local councils, boards, committees or other types of groups to support community participation. In particular, trust by the community, inclusion of the broader community in decision making and transparency were some of the factors that helped communities work towards a common goal.

### For whom communities act

Elements of *for whom communities act* that were detailed in articles documenting rich experiences of community participation in service delivery and governance include leadership and conflict resolution. These elements were considered important in determining the interests served by the community’s collective action and social processes.

#### Leadership

Eight articles discussed the presence of an influential, strong champion who can advocate for the uptake and continuation of the program [17, 20, 31, 43, 53, 58, 64, 78].

In addition, we examined how community leaders represented different group interests across the community and how they exercised power and decision making collectively. Only ten articles were found to demonstrate these leadership characteristics. The most notable (and positive) example is the introduction of pagoda managed equity funds in Cambodia where leadership through health committees, which had been earlier graded as fair, improved considerably after the introduction of the equity funds, and volunteers were reported to actively promote financial access to health services for the poor [18].

In contrast, five articles detailed the lack of substantial connections between the leadership and the community or that community-based leaders had passive or limited decision making authority. In Uganda, the lack of communication between community leaders and the broader community, impeded the appropriate functioning of participatory planning mechanisms [30], highlighting how management of information is a key aspect of effective community leadership.

Numerous articles noted the limitations of health committees or boards to support effective community representation. For example, in an accountability intervention in Coast Province, Kenya using health facility committees, only 4 % of community members had interacted with a committee member regarding health facility management issues, and there were some reports of mistrust between community members and committee members [56]. In another example, an urban health intervention in Dar es Salaam utilized health boards with community representation; however, community representatives on these boards have insufficient input from the wider community and therefore, one can assume do not serve as an adequate platform for enhancing the voice of community members [64]. A health sector reform project in Pakistan is assumed to have not positively impacted the voice of community members due to the inability of the project to operationalize community participation mechanisms, such as village health committees [25].

#### Conflict resolving

We reviewed studies for whether interventions included strategies, formal or informal, to fairly solve interpersonal conflict among those individuals engaged in the intervention. Only five articles discussed strategies to solve conflict [22, 27, 30, 48, 56]. Peer researchers, as part of a community-based research project involving drug users in Bangkok, Thailand, reported improved conflict solving abilities, including openly negotiating cash remuneration with their colleagues from academic settings [48]. A study of health facility committees in the Coastal Province of Kenya found that facility management nurses often resolved conflicts between health workers and community members serving on the committees; authors, however, did not provide details on how extensive this approach had been [56].

As discussed earlier, interventions seeking to enhance social equity resulted in many instances of social tension and conflict. An exploration into women’s health groups in Mexico revealed that women had to renegotiate relationships with their husbands, family members and neighbors in order to openly participate in the groups [22]. A study of participatory health planning and prioritization in Uganda found that there was conflict between youth and adults in decision-making processes, with adult men complaining about the right of youth to participate in these processes, and the youth limiting their participation due to their fear of adults [30]. Conversely, conflict with regards to community participation in rural Mali was averted in one site as community members were barely engaged in the intervention in the first place [27].

## Discussion

### Summary of evidence

Community capability is the combined influence of a community’s social systems and collective resources that can be applied to address community problems and broaden community opportunities. Previous reviews and efforts to measure it have developed a long list of overlapping concepts. We simplified the framing of community capability as consisting of three domains which synergistically support community empowerment: *what communities have; how communities act,* and *for whom communities act.*

When considering *what communities have,* most articles discussed in order of frequency: external links, gaining skills and information and accruing physical assets. External linkages had three broad purposes (to increase delivery and utilization of services, to improve accountability of services, and to support higher level planning), which often overlapped. These linkages were powerful ways to leverage community representation and address community needs, but only if communities were supported by networks and included in key mechanisms enabling and valuing bottom up planning. As noted previously, government mandates opening spaces for community participation are a key contextual factor, alongside the presence of social movements and the specific histories of collective experience and action specific to each community [2].

Relatively few articles referred to the development of and control over financial assets as a key resource supporting community capability. This gap in the community participation for health literature regarding communities gaining and managing financial resources may also reflect a publication bias. Health initiatives that are externally funded may be less likely to emphasize financial resources and may also be more likely to be written up for publication. Articles in the review mentioned physical, information and financial assets in a descriptive fashion, with few delving into issues of whether communities had control over these resources and how they were governed. Despite this weakness, many communities undoubtedly gained valuable resources, information, skills and linkages through these participatory health systems interventions. The importance of these gains to participants highlights the extensive pragmatic and material needs facing marginalized communities and the ongoing necessity for future initiatives to further both psycho-social empowerment and material empowerment [11, 79].

With *how communities act* articles discussed challenges of ensuring breadth of participation and explicit strategies to ensure inclusiveness. Various mechanisms supported breadth of participation such as decision making through public forums, community-wide meetings or community dialogues or even more explicit measures prioritizing inclusion of marginalized groups. Nonetheless, inclusiveness required substantial community leadership to overcome potential conflicts that maintained social divisions within communities. In contrast to breadth of participation, very little was reported about cohesiveness and efficacy despite their importance in motivating and strengthening community initiatives. Community efficacy in particular hinged on breadth of participation, trust and transparency. These latter elements take time to nurture, leading to timelines that are often contrary to funding deadlines [80–83].

When reviewing *for whom communities act,* the importance of leadership in terms of strong champions was mentioned more frequently in contrast to conflict resolution, but these governance elements of community capability were overall not well represented in the literature. This is a disconcerting gap, especially when one considers managing power to be at the heart of community participatory processes. While there were positive examples of community leadership representing different group interests across the community and exercising power and decision making collectively, there were also numerous examples of leadership and mechanisms meant to engender community voice that failed to represent community needs.

Important synergies were found across these elements of community capability. Leadership played a crucial role in information sharing and conflict resolution, with the latter having an important role in ensuring social equity. Tangible success in communities organizing local transport systems spilled over into greater cohesiveness and motivation supporting lay health worker associations and savings funds. Information and skills building were crucial in supporting community participation, critical thinking, problem solving and ownership. Some articles detailed synergies between the creation of physical or financial assets and other community activities and abilities. Tangible rewards provide powerful motivation for collective endeavors. These linkages highlight the dynamic complexity and strength of building community capability, and cautions against initiatives that may view it as a simplistic linear process between inputs and outputs.

Each element of community capability had positive examples, but also numerous examples detailing limitations from health systems research efforts to support community participation. Breadth of participation and the institutional process that support them were mentioned the most across articles that discussed community characteristics that support collective action. Sometimes the experiences described could be viewed as either positive or negative depending on one’s perspective. Half of these articles reported improving social equity with regards to gender, class, ethnicity, minority group status or age. For those who see community participation as a central process for ensuring more equitable health systems more articles should have measured or discussed social equity dimensions. For those more focused on health outcomes, the extent to which social equity was represented may be interpreted as a positive finding.

The concept of community capability overlaps with the “health enabling community” or “competent community,” often applied to community interventions for HIV prevention and management [84]. As with the concept of community capability, the theory of a “competent” and “health enabling” community emphasizes the importance of the community as a context for empowerment and the development of positive social identities, which ultimately support positive health behaviors [85, 86]. One key aspect of the health enabling community is social capital, wherein social relationships generate resources, which include a sense of belonging, trust in social institutions, reciprocity, social influence, access to new information, the enforcement of social sanctions, that support community capability to uptake new behaviors and sustain the changes entailed [87, 88]. We found that health systems research on community capability engaged far more with the “bridging” aspect of social capital—the need to build social connections between communities and outside stakeholders who bring new ideas and resources. Strengthening “bonding” social capital—the within-community relationships of reciprocity and trust—was less clearly discussed. While several tools have been developed to measure social capital in LMICs [89, 90], systematic efforts to build social capital and documentation of these efforts require further attention [88].

As much as health systems initiatives seek to work with communities and strengthen their capabilities, communities are diverse and human agency can be ingeniously autonomous or unpredictable. Unlocking community capabilities enables communities to make use of their unique social systems and resources in ways that may not always align with outsider expectations [91]. Policymakers and program implementers must consider what “community participation” means in terms of control over agendas, resources, processes and outcomes as well as in terms of who counts as the community [91]. The studies reviewed here highlight that participatory health system interventions at the community level play out in specific contexts; interventions must navigate pre-existing power dynamics and the additional tensions created by introducing new resources and expectations. Interrogating assumptions about the social processes that underpin how communities participate in health systems interventions is critical to developing more realistic expectations, adequate resources and supportive principles of collaboration to facilitate community empowerment and broader social development. This makes learning from the full range of experiences both positive and negative important, and makes the documentation issues of governance whether related to control over resources; inclusiveness; representative and democratic leadership and conflict resolution, all the more important.

### Limitations

While there is an evolving body of work on the quantitative measurement of community capability, with varied components and scales, this has not filtered through to those supporting community participation more broadly. In addition, despite the general acknowledgement of the importance of understanding the social processes and conditions that represent community capability is critical for initiatives that support community participation, we found that the quality of description by articles of these elements was very low. Studies may have considered and measured many of the elements central to this review, but not published them in the articles that were in our review. Several of the concepts we examined overlap. While we did present quantifications to characterize the literature, a significant portion of the decision-making, abstraction and interpretation is subjective. Throughout the review, we not only convened regular group discussions to evaluate our understanding of the subject, but also documented our deliberations.

## Conclusions

Although the development of scales measuring community capability through numerous elements continues to evolve [4, 9, 92–94], health systems research engaged with community participation remains relatively untouched by these developments. Our review found the extent of information related to community capability reported by health systems research articles with rich accounts of community participation to be very low and often of poor quality. Having a simpler framework to define community capability may help those who are not specialists in community capability better recognize it, support it in their interventions and measure it in their evaluations. Our framework includes tangible gains, skills and material resources that communities need to sustain community participation, as well as the more intangible social processes related to cohesiveness, trust and efficacy. Even with a simplified framework, significant gaps in the literature need to be addressed. While attention to equity across the articles was variable, social hierarchies are significant, particularly for marginalized groups. More attention needs to be paid in particular to governance elements, leadership and conflict resolution if power relations that inhibit marginalized groups are to be overcome. Strengthening community capability is critical to ensuring that community participation does lead to empowerment and shift the balance of power in building equitable health systems and improving health outcomes for those who have been excluded from these processes for too long.

## Abbreviations

AIDS: Acquired immunodeficiency syndrome
APOC: African Program for Onchocerciasis Control
CSOs: Civil society organizations
DOTS: Directly observed treatment short course
HIV: Human immunodeficiency virus
LMICs: Low- and middle-income countries
